# Cabergoline possesses a beneficial effect on blood-brain barrier (BBB) integrity against lipopolysaccharide (LPS)

**DOI:** 10.1080/21655979.2021.1987066

**Published:** 2021-10-21

**Authors:** Lina You, Haidong Jiang

**Affiliations:** a Department of Gerontology, Traditional Chinese medicine hospital of Jiulongpo District in Chongqing, Chongqing, 400080, China; b Chongqing Infectious Disease Medical Center, Chongqing, 400080, China

**Keywords:** Cabergoline, blood-brain barrier, sepsis, DKK3, LPS

## Abstract

Sepsis is a disease induced by severe systemic inflammation and contributes to multiple acute organic dysfunctions. It is reported that disrupted blood-brain barrier (BBB) integrity is involved in sepsis-associated encephalopathy (SAE), which can be alleviated by repairing the damaged tight junction structure. Cabergoline is a specific dopamine D2 receptor agonist developed to treat Parkinson’s disease and hyperprolactinemia and is reported to exert promising anti-inflammatory properties. The present study aimed to explore the beneficial effect of Cabergoline for the treatment of sepsis. In the animal experiments, mice were separated into 4 groups: sham, LPS (5 mg/kg), Cabergoline (0.1 mg/kg/day), and Cabergoline+LPS. We found that the increased neurological deficits, disrupted BBB integrity, elevated production of inflammatory factors, and declined expression level of zonula occludens-1 (ZO-1) were observed in lipopolysaccharide (LPS)-treated mice, all of which were significantly reversed by the administration of Cabergoline. In the *in vitro* model, human brain microvascular endothelial cells (HBMECs) were challenged with 1 µg/mL LPS in the presence or absence of Cabergoline (10, 20 μM) for 24 hours. The elevated cell permeability P_app_ value of fluorescein disodium across the HBMECs monolayer and declined trans-endothelial electrical resistance (TEER) in the LPS-treated HBMECs were significantly alleviated by Cabergoline, accompanied by the upregulation of ZO-1. In addition, wnt1 and β-catenin were found downregulated, which was reversed by Cabergoline. Importantly, the protective benefits of Cabergoline were all abolished by the overexpression of Dickkopf 3 (DKK3). Taken together, our data reveal that Cabergoline possessed a protective effect on BBB integrity against LPS.

## Introduction

Sepsis is a systemic inflammatory reaction induced by infection, which contributes to acute organic dysfunction [1–3]. Sepsis-associated encephalopathy (SAE) is a kind of diffuse brain injury (DBI) induced by sepsis and is mainly characterized by secondary consciousness and cognitive dysfunction [4]. As a complication of sepsis, the morbidity of SAE ranges from 7% to 81% [5]. At the early stage of sepsis, brain dysfunction is observed and significantly increases the mortality of sepsis patients [6–9]. SAE is a non-organic brain dysfunction induced by multiple factors and its pathological mechanism includes brain microcirculation disorder, neurotransmitter changes, neuroinflammation, and blood-brain barrier (BBB) function damage, among which BBB dysfunction is the central step for the development of SAE [10–12]. Currently, there are no definitive therapies for SAE.

The BBB is a selective semipermeable membrane located between the brain tissues and the blood, which plays an important role in regulating brain homeostasis and maintaining normal brain function [13]. It is composed of brain microvascular endothelial cells (BMECs), tight junctions (TJ), a basement membrane, perithelial cells, and astrocytes foot processes [14]. The peripheral inflammatory signals are introduced into the central nervous system (CNS) through the periventricular apparatus and vagus nerve. This causes glial cells activation, after which they release excessive proinflammatory factors, such as interleukin (IL)-1β, tumor necrosis factor (TNF)-α, nitric oxide (NO), and prostaglandins, which induces the production of complements, cytokines, and chemokines around the BBB. The brain microvascular endothelial cells (BMECs) will be damaged, swollen astrocytes and perithelial cells will be observed, and the permeability of the BBB will be increased, finally contributing to the disruption of the BBB and brain injury [15].

It is reported that significantly increased protein concentration in the cerebrospinal fluid and brain edema have been observed in sepsis patients [16,17], indicating severe BBB disruption. TJs among BMECs play a critical role in regulating BBB permeability and function, and they are composed of transmembrane proteins, such as occludins, claudins, and cytoplasmic proteins [18]. These TJs, together with BMECs, cytoskeletal actions, and myosin, maintain the stability of the BBB structure. Therefore, TJs might be an important target for BBB protection in the treatment of SAE.

**Figure 1.**
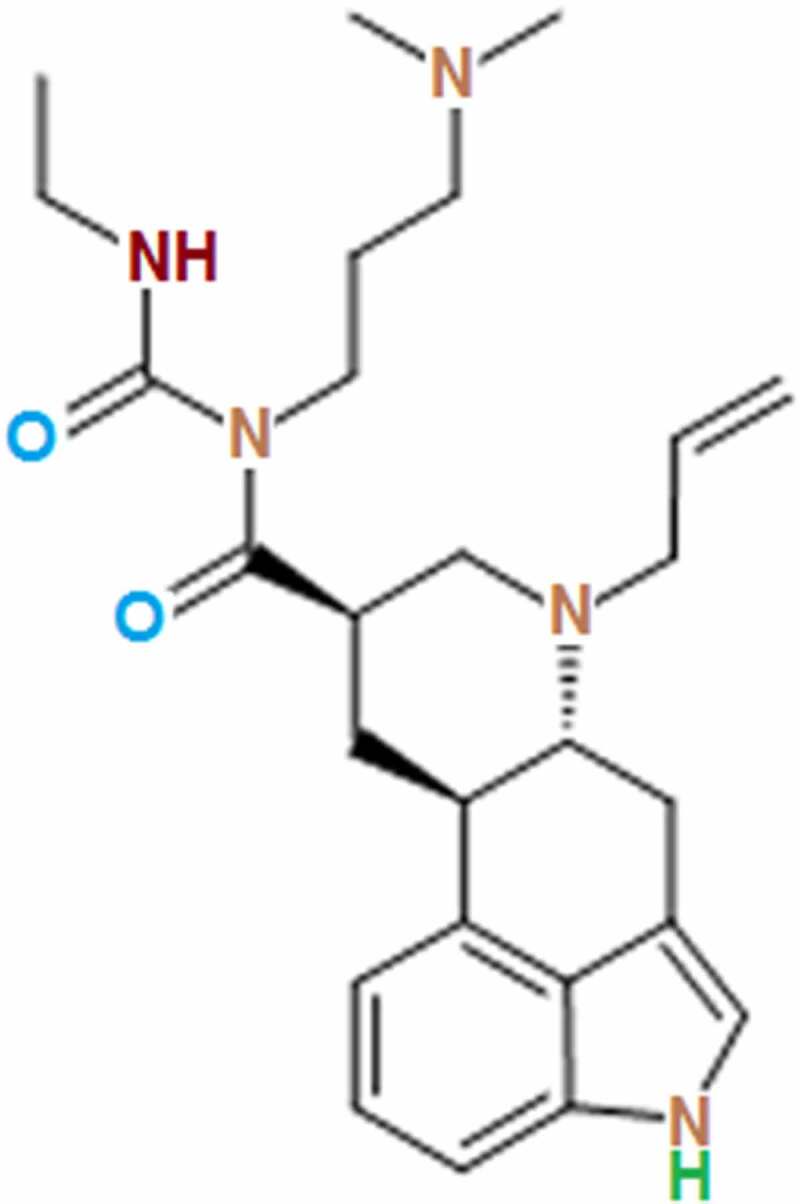
Molecular structure of Cabergoline

Cabergoline (Figure 1) is an artificial Ergoline derivative developed by Farmitalia CarloEr-ba for the treatment of Parkinson’s disease and hyperprolactinemia. It inhibits lactation by selectively targeting the dopamine D2 receptor to suppress the function of prolactin. Recently, it has been reported to exert anti-inflammatory effects when treating Parkinson’s disease [19]. Interestingly, the dopamine D2 receptor has been found to be expressed in endothelial cells, and we speculate that Cabergoline might exert protective benefits in brain vessels [20]. The present study aims to explore the therapeutic effect of Cabergoline on SAE by investigating its protective property in a disrupted BBB against lipopolysaccharide (LPS).

## Materials and methods

### Animals and sepsis modeling

For the animal experiments, forty male and female C56BL/6 mice aged 8 weeks were purchased from Zhejiang University Laboratory Animal Center and randomly divided into 4 groups averagely with the number of male and female mice similar in each group. In the Sham group, animals were injected with normal saline (i.p.) 3 times a day (0, 12 h, and 24 h). In the Cabergoline group, mice were orally dosed with Cabergoline at a dosage of 0.1 mg/kg/day for 3 days [21]. In the LPS group, mice were injected with 5 mg/kg LPS (i.p.) 3 times a day (0, 12 h, and 24 h) [22]. Lastly, in the Cabergoline + LPS group, mice were injected with 5 mg/kg LPS (i.p.) 3 times a day on Day 1 and orally dosed with Cabergoline at a dosage of 0.1 mg/kg/day for 3 days.

### Measurement of neurological deficits

The neurological deficits of mice were evaluated using Longa’s five-point scale method and with the following rules determining the scores: 0, no deficits; 1, failure to extend right forepaw; 2, circling to right; 3, falling to right; 4, unconscious and disable to walk spontaneously.

### Determination of the BBB integrity with sodium fluorescein

Mice were injected (i.v.) from the tails with 100 mg/kg sodium fluorescein dye and then anesthetized using a mixture of 100 mg/kg Ketamine and 10 mg/kg Xylazine. Following the removal of brain tissues, samples were homogenized using the cold PBS buffer, followed by protein precipitation using 30% trichloroacetic acid. Then, samples were centrifugated at 14,000 g for 10 min and the supernatant was collected, followed by detecting the absorbance at 440/525 nm using the spectrophotofluorometer (HACH, Colorado, USA) to determine the concentration of sodium fluorescein.

### Real-time PCR assay

Homogenates of brain tissues and endothelial cells were used to extract total RNA using the Trizol solution (Life, New York, USA), followed by being transcribed into cDNAs with a Moloney Murine Leukemia Virus Reverse transcriptase (Fermentas, Vancouver, Canada). The ABI 7500 real-time thermocycler (Applied Biosystems, California, USA) was used to perform the PCR reaction and the LightCycler1.5 (Applied Biosystems, California, USA) and the AceQ qPCR SYBR Green Master Mix (Vazyme, Nanjing, China) were used to conduct the real-time PCR. Finally, the 2^−ΔΔCt^ method was utilized to determine the expression level of genes by normalized to glyceraldehyde 3-phosphate dehydrogenase (GAPDH). The following primers were used: IL-6, Fwd: 5ʹ- TGCCTTCTTGGGACTGATGC-3ʹ, Rev: 5ʹ-GCAAGTGCATCATCGTTGTTC-3ʹ; TNF-α, Fwd: 5ʹ- GACGTGGAACTGGCAGAAGA-3ʹ, Rev: 5ʹ-GGCTACGGCTTGTCACTCG-3ʹ; DKK3, Fwd: 5ʹ-CTCGGGGGTATTTTGCTGTGT-3ʹ, Rev: 5ʹ-TCCTCCTGAGGGTAGTTGAGA-3ʹ; ZO-1, Fwd: 5ʹ-CCGCTAAGAGCAC AGCAAT-3ʹ, Rev: 5ʹ-CATTGCAACTCGGTCATTTT-3ʹ; GAPDH, Fwd: 5ʹ-TGACCTCAACTACATGGTCTACA-3ʹ, Rev: 5ʹ- CTTCCCATTCTCGGCCTTG-3ʹ.

### Immunostaining assay

Brain tissues were isolated from each mouse and fixed in 2% formaldehyde solution, followed by being embedded in paraffin and cut into 4-μm-thick sections. After being deparaffinated and rehydrated, sections were incubated with 3% H_2_O_2_ for 15 min and blocked with 5% BSA in TBST for 30 min. Then sections were incubated with the primary antibody against ZO-1 (1:200, Abcam, Cambridge, UK), followed by being incubated with HRP-conjugated secondary antibody (Abcam, Cambridge, UK). Lastly, images were taken using a light microscope (Olympus, Tokyo, Japan).

### ELISA assay

The concentrations of IL-6 and TNF-α in brain tissues were detected using the ELISA assay (Elabscience, Wuhan, China). Briefly, the supernatant was collected from the homogenates of brain tissues and seeded on a 96-well plate together with the standards to be incubated for 1 h. After removing the medium, the conjugate solutions were added for 30 min incubation, and then the TMB solution was added for 15 min incubation. Lastly, the reaction was ended using the stop solution and the absorbance at 450 nm was measured using the microplate reader (Biotek, Vermont, USA).

### Cell culture, treatment, and adenovirus transduction

Human BMECs (HBMECs) were purchased from ATCC (ATCC, California, USA), treated with 10 and 20 μM Cabergoline, and incubated in EGM-2 Endothelial Cell Growth Medium-2 BulletKit (Lonza, Switzerland) containing 10% FBS under the condition of 5% CO_2_ and 37°C [23,24]. To establish the DKK3-overexpressed HBMECs, cells were transfected with adenovirus containing pcDNA3.1-DKK3 plasmids (Genscript, Nanjing, China) (Invitrogen, California, USA). After incubation for 48 hours, the efficacy of transduction was confirmed using the Western blotting assay.

### Endothelial permeability

The cell permeability P_app_ value of fluorescein disodium across HBMECs was detected to evaluate the endothelial permeability. In brief, cells were planted into the upper chamber of the transwell (Corning, New York, USA) at a density of 2 × 10^5^ cells/well, followed by adding the blank medium into the lower chamber. After incubating for 7 days, the transwell was washed using the serum-free medium and 10 μg/mL Na-Flu was added into the upper inserts and the serum-free medium was added into the lower chamber. After incubation for 1 h, the medium in the lower chamber was collected at different time points to detect the absorbance at 440/525 nm using the spectrophotofluorometer (HACH, Colorado, USA). The permeability was calculated using the Artursson formula: P_app_ = (dQ/dt)×1/(A*C_0_*60). The value of dQ/dt represented the past Na-Flu per minute. A represented the superficial area of the chamber. C_0_ represented the initial concentration of Na-Flu.

### Trans-endothelial electrical resistance (TEER) assay

In brief, cells were planted into the upper chamber of the transwell (Corning, New York, USA) at a density of 2 × 10^5^ cells/well, followed by adding the blank medium into the lower chamber. The TEER value was then measured using the Maestro Z (Axion BioSystems, Atlanta, USA) after incubating for various time intervals.

### Western blot analysis

Total proteins were isolated from HBMECs using the lysis buffer and were quantified with a BCA kit (Takara, Tokyo, Japan). Proteins were loaded and separated with 12% SDS-PAGE, followed by being transferred to the PVDF membrane (Takara, Tokyo, Japan). After being incubated with 5% skim milk, the membrane was incubated with the primary antibody against ZO-1 (1:800, Abcam, Cambridge, UK), DKK3 (1:800, Abcam, Cambridge, UK), wnt1 (1:800, Abcam, Cambridge, UK), and β-catenin (1:800, Abcam, Cambridge, UK), followed by being incubated with the secondary antibody (1:800, Abcam, Cambridge, UK) at room temperature for 1.5 hours. Finally, the ECL solution was used to visualize the bands, which were quantified by the Image J software.

### Statistical analysis

Data were expressed as standard error (S.E.) and the data analysis was conducted using the GraphPad software. The Student’s t-test was used to analyze data between 2 groups and the ANOVA method was used for the analysis among groups, followed by Tukey’s post-hoc test. P < 0.05 was considered a significant difference.

## Results

Using both *in vivo* mice and *in vitro* cells models, we investigated the effects of Cabergoline on LPS- induced BBB permeability. We tested BBB permeability, expressions of ZO-1, and inflammatory mediators in LPS- treated mice. Furthermore, we examined endothelial monolayer permeability, and the expressions of ZO-1 and DKK3 in LPS- treated HBMECs.

### Cabergoline improved neurological deficit in LPS-treated mice

After different strategies, the total neurological score was determined on each mouse. We found that the total neurological score in the control and Cabergoline groups was 0 in both, which was significantly elevated to 2.5 in the LPS group. After treatment with Cabergoline, the total neurological score was dramatically decreased to 1.6, indicating a promising protective effect of Cabergoline on the neurofunction in LPS-treated mice.

### Cabergoline reduced LPS-induced increase in BBB permeability in mice brains

**Figure 2.**
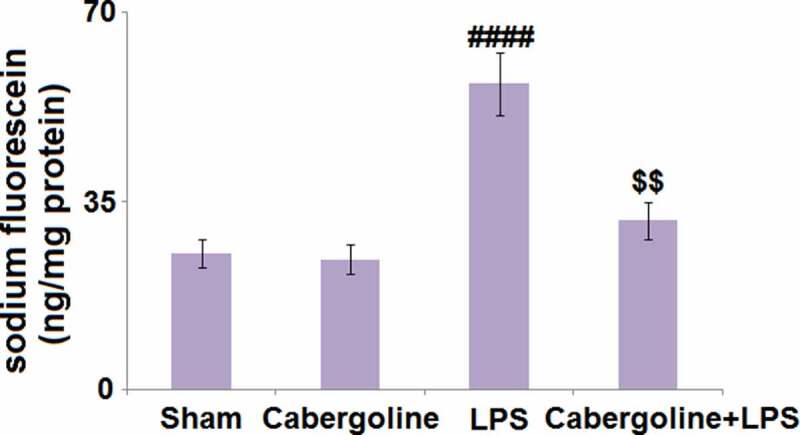
Cabergoline reduces LPS-induced increase in blood–brain barrier permeability in mice brains. Blood–brain permeability was evaluated by intravenous injection sodium fluorescein (####, P < 0.001 vs. vehicle group; $$, P < 0.01 vs. LPS group, n = 9–10)

The BBB plays an important role in protecting the brain tissue under the stimulation of LPS. Compared to the Sham group, the sodium fluorescein was slightly changed from 25.3 ng/mg protein to 24.2 ng/mg protein in the Cabergoline group and dramatically elevated to 56.8 ng/mg protein by LPS. It was then greatly declined to 31.4 in the LPS+Cabergoline group (Figure 2), indicating that the LPS-induced increase in BBB permeability in mice brains was significantly alleviated by Cabergoline.

### Cabergoline increased the expression of ZO-1 in LPS-challenged mice brains

**Figure 3.**
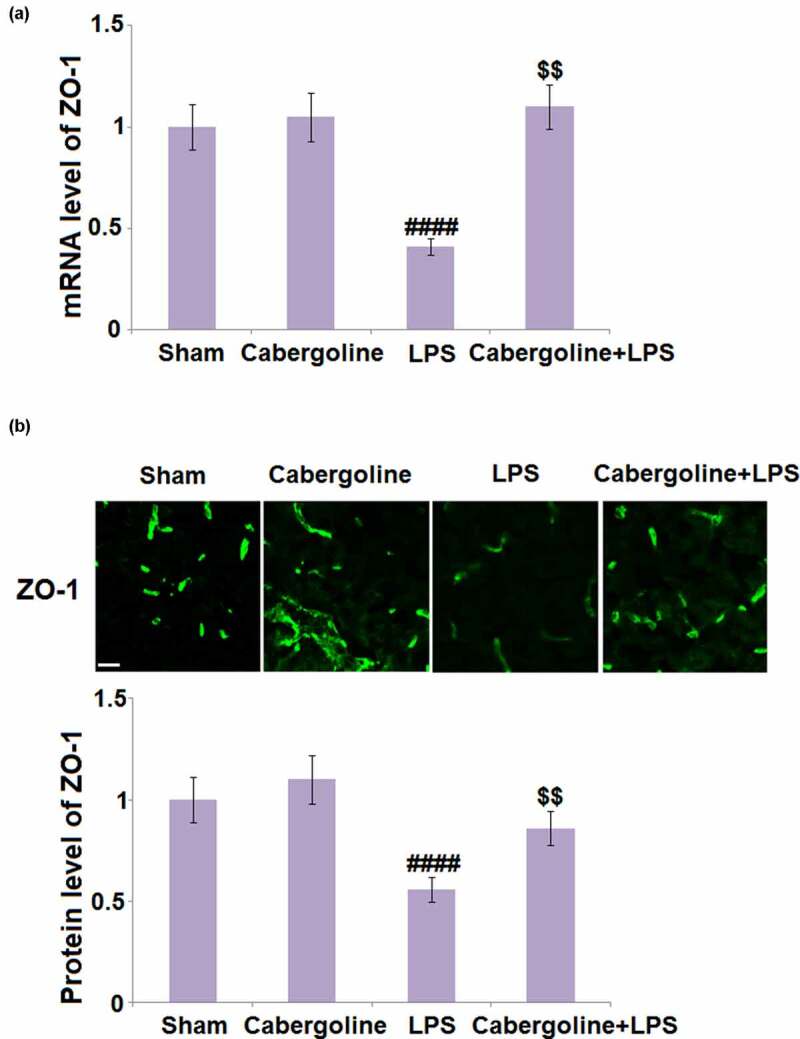
Cabergoline increases expression of ZO-1 in LPS-challenged mice brains. (a) mRNA level of ZO-1; (b) Protein level of ZO-1 determined by immunostaining. Scale bar, 50 μm (####, P < 0.001 vs. vehicle group; $$, P < 0.01 vs. LPS group, n = 9–10)

ZO-1 is an important TJ protein that regulates the permeability and function of the BBB [25]. As shown in Figure 3(a,b), compared to the sham group, the expression level of ZO-1 was unchanged in the Cabergoline group but significantly decreased in the LPS group. This was greatly reversed in the LPS + Cabergoline group, indicating a promising alleviation by Cabergoline on downregulated ZO-1 in LPS-treated mice.

### Cabergoline inhibited the production of inflammatory mediators which were elevated by LPS in brain vessels

**Figure 4.**
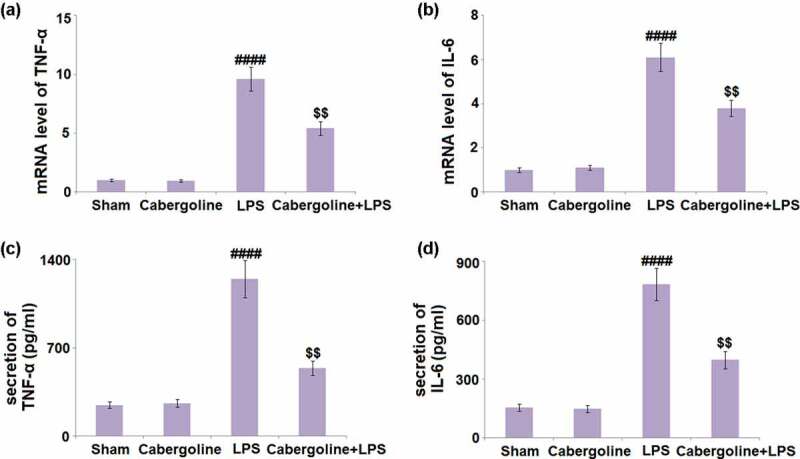
Cabergoline inhibits the production of inflammatory mediators in LPS- challenged mice brain vessels. (a) mRNA level of TNF-α; (b) mRNA of IL-6; (c). Secretion of TNF-α; (d) Secretion of IL-6 (####, P < 0.001 vs. vehicle group; $$, P < 0.01 vs. LPS group, n = 9–10)

Excessive production of inflammatory factors is a critical pathological symptom following an increased BBB permeability [26]. We further detected the concentrations of inflammatory factors in brain tissues. We found that the gene expression levels of TNF-α and IL-6 (Figure 4(a-b) in the Cabergoline group remained unchanged compared to the Sham group, but were significantly upregulated by the stimulation with LPS. When Cabergoline was introduced, TNF-α and IL-6 were greatly downregulated. The production of TNF-α (Figure 4(c)) in the Sham, Cabergoline, LPS, and LPS + Cabergoline group was 245.8, 261.2, 1247.5, and 538.6 pg/mL, respectively. In addition, the secretion of IL-6 was slightly changed from 153.4 to 148.1 pg/mL and then significantly elevated to 784.2 pg/mL in the LPS group. Following treatment with Cabergoline, it was pronouncedly repressed to 397.5 pg/mL (Figure 4(d)). These data reveal a promising anti-inflammatory effect of Cabergoline in LPS-treated mice.

### Cabergoline attenuates HBMECs monolayer permeability induced by LPS in HBMECs

**Figure 5.**
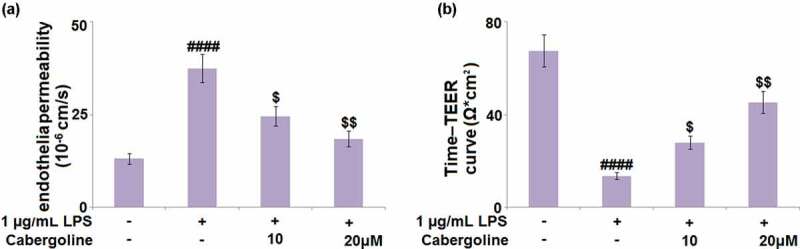
Cabergoline attenuates HBMECs monolayer permeability induced by LPS in HBMECs. Cells (1x10^5^ cells/well) were seeded onto 24-well trans-well inserts and then challenged with 1 µg/mL LPS for 24 h in the presence and absence of Cabergoline (10, 20 μM). (a) The Cell permeability P_app_ value of fluorescein disodium across HBMECs monolayer was evaluated. (b) TEER of the integrity of HBMECs monolayer (####, P < 0.001 vs. vehicle group; $, $$, P < 0.05, 0.01 vs. LPS group, n = 5–6)

Cells (1x10^5^ cells/well) were seeded onto 24-well trans-well inserts and then challenged with 1 µg/mL LPS for 24 h in the presence or absence of Cabergoline, followed by evaluating the endothelial permeability using the P_app_ value and TEER assay. Compared to the control, the P_app_ value (Figure 5(a)) was significantly elevated from 13.1 × 10^−6^ cm/s to 27.5 × 10^−6^ cm/s by the stimulation with LPS, then greatly repressed to 24.6 × 10^−6^ cm/s and 18.5 × 10^−6^ cm/s by 10 and 20 μM Cabergoline, respectively. In addition, after treatment with LPS for 24 h in the presence or absence of Cabergoline, the peak TEER in the control, LPS, 10 and 20 μM Cabergoline groups was 67.5, 13.5, 27.9, and 45.3 Ω*cm^2^, respectively (Figure 5(b)). These results indicate that the increased HBMECs monolayer permeability induced by LPS was significantly ameliorated by Cabergoline.

### Cabergoline restored the expression of ZO-1 in LPS-challenged HBMECs

**Figure 6.**
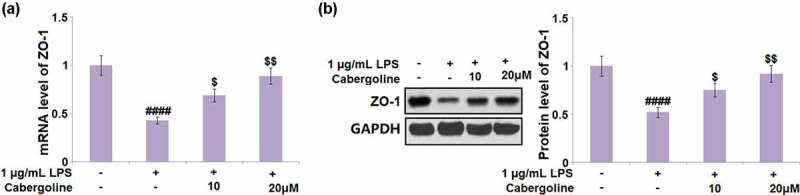
Cabergoline restored the expression of ZO-1 in LPS-challenged HBMECs. Cells were challenged with 1 µg/mL LPS in the presence and absence of Cabergoline (10, 20 μM) for 24 hours. (a) mRNA level of ZO-1; (b) Protein level of ZO-1 (####, P < 0.001 vs. vehicle group; $, $$, P < 0.05, 0.01 vs. LPS group, n = 5–6)

Consistent with the *in vivo* experiments, ZO-1 expression by HBMECs was determined following different treatment strategies. We found that the declined expression level of ZO-1 in LPS-treated HBMECs was significantly elevated by the introduction of 10 and 20 μM Cabergoline (Figure 6(a-b)).

### Cabergoline reduced the expression of DKK3 and activated the Wnt/β-catenin pathway in LPS-challenged HBMECs

**Figure 7.**
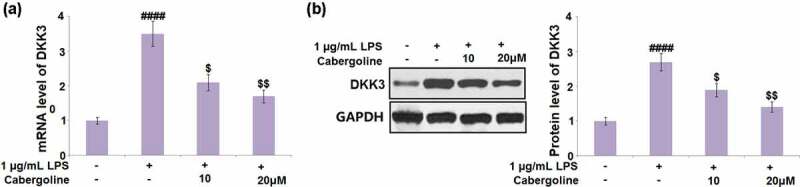
Cabergoline reduced the expression of DKK3 in LPS-challenged HBMECs. (a). mRNA level of DKK3; (b). Protein level of DKK3 (####, P < 0.001 vs. vehicle group; $, $$, P < 0.05, 0.01 vs. LPS group, n = 5–6)

**Figure 8.**
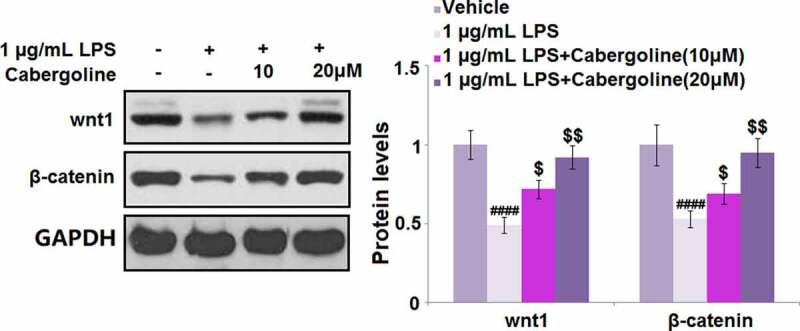
Cabergoline activated the Wnt/β-catenin pathway in LPS-challenged HBMECs. Protein levels of wnt1 and β-catenin were determined (####, P < 0.001 vs. vehicle group; $, $$, P < 0.05, 0.01 vs. LPS group, n = 5–6)

It is reported that the permeability induced by ethanol in brain endothelial cells can be reversed by the activation of the Wnt/β-catenin pathway [27] and DKK3 is its natural inhibitor [28]. We suspected the possible association between the protective effect of Cabergoline and the activation of the Wnt/β-catenin pathway, and the inhibitory effect on DKK3 expression. As shown in Figure 7(a-b), we found that the expression level of DKK3 was significantly elevated in the LPS group, but greatly repressed by 10 and 20 μM Cabergoline. In addition, the downregulated Wnt and β-catenin in LPS-treated HBMECs were dramatically upregulated by 10 and 20 μM Cabergoline (Figure 8). These data reveal that Cabergoline might protect the BBB by activating the DKK3-mediated Wnt/β-catenin pathway.

### Cabergoline mediates LPS-induced HBMECs monolayer permeability via DKK3

**Figure 9.**
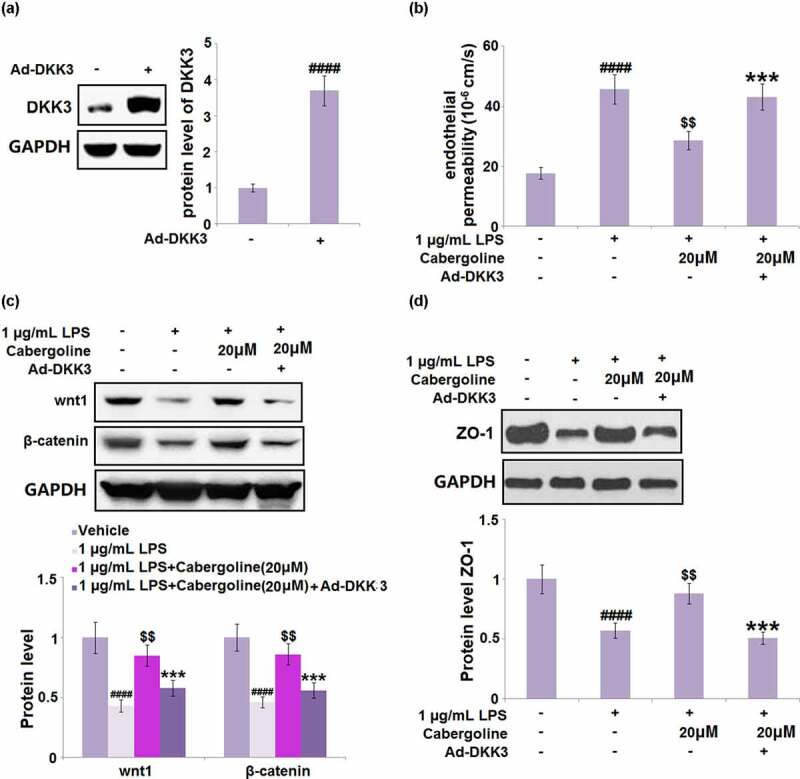
The effects of Cabergoline against LPS-induced HBMECs monolayer permeability are mediated by DKK3. HBMECs were transfected with DKK3-overexpressing adenovirus or vehicle and then challenged with 1 µg/m L LPS h in the presence and absence of Cabergoline (20 μM) for 24. (a) protein level of DKK3 was determined; (b) The Cell permeability P_app_ value of fluorescein disodium across HBMECs monolayer was evaluated; (c). Protein level of wnt1 and β-catenin; (d) Protein level ZO-1 was determined (####, P < 0.001 vs. vehicle group; $$, P < 0.01 vs. LPS group; ***, P < 0.005 vs. LPS+ Cabergoline group, n = 5–6)

To further confirm that the regulatory effect of Cabergoline on the Wnt/β-catenin pathway was associated with the inhibition of DKK3, HBMECs were transfected with DKK3-overexpressing adenovirus or vehicle, and then challenged with 1 µg/mL LPS in the presence or absence of Cabergoline (20 μM) for 24 h. The transfection efficacy was confirmed by the results shown in Figure 9(a). We found that the P_app_ value (Figure 9(b)) of fluorescein disodium across the HBMECs monolayer was increased from 17.7 × 10^−6^ cm/s to 45.7 × 10^−6^ cm/s in the LPS group, which was greatly suppressed to 28.6 × 10^−6^ cm/s by Cabergoline. After the transfection of pcDNA3.1-DKK3, the P_app_ value was reversed to 43.11 × 10^−6^ cm/s. In addition, the downregulated Wnt and β-catenin (Figure 9(c)) in LPS-treated HBMECs were dramatically reversed by Cabergoline but further downregulated in the DKK3-overexpressed HBMECs. Lastly, the declined expression level of ZO-1 (Figure 9(d)) in LPS-treated HBMECs was pronouncedly elevated by Cabergoline, which was abolished by the transfection of pcDNA3.1-DKK3. These results indicate that the protective effect of Cabergoline on LPS-induced permeability in HBMECs is associated with the inhibition of DKK3.

## Discussion

In this study, we investigated the potential benefits of Cabergoline in BBB integrity maintenance against LPS. We found that LPS treatment increased neurological deficits, disrupted BBB integrity, elevated the production of inflammatory factors, and declined the expression level of ZO-1, all of which were significantly reversed by the administration of Cabergoline. In the *in vitro* model, the elevated cell permeability P_app_ value of fluorescein disodium across the HBMECs monolayer and declined TEER in LPS-treated HBMECs were significantly alleviated by Cabergoline, accompanied by the upregulation of ZO-1. In addition, Wnt1 and β-catenin were found downregulated, which was reversed by Cabergoline. Importantly, the protective benefits of Cabergoline were all abolished by the overexpression of DKK3.

The permeability of the BBB is reportedly changed during the development of sepsis, inducing a series of pathological and functional changes in brain tissues, including edema [15]. In the animal sepsis model, the expression levels of inflammatory factors, such as TNF-α, IL-1, and IL-6, are significantly elevated in brain tissues, accompanied by the increased cerebral water content and brain permeability to blood dyes [29]. In the present study, we found that in both the animal model and the *in vitro* endothelial monolayers model, LPS significantly increased permeability, which is consistent with previous reports [30,31]. After the treatment with Cabergoline, the increased permeabilities of the BBB and endothelial monolayers were significantly reversed, indicating that the disrupted BBB by LPS could be repaired by Cabergoline. In addition, the increased production of pro-inflammatory factors was observed in the brain vessels of LPS-treated animals. It was then dramatically repressed by Cabergoline, indicating that inflammation in brain tissues induced by the disruption of the BBB was significantly alleviated by Cabergoline.

TJs are important structures regulating the permeability of the BBB [32]. *In vitro* experiments indicate that the TEER of HBMECs monolayers is decreased and their permeability to dextran molecules enhanced [33], which is consistent with the results observed in the *in vitro* experiments in the present study. Under the scanning electron microscope, TJs in HBMECs monolayers are observed to be disrupted by TNF-α, which results in the shuttling of horseradish peroxidase among HBMECs [33]. The normal BBB permeability is determined by the interaction between TJ proteins and actins through the connection by ZO-1 [33]. When the BBB permeability changes, the expression levels of TJ-related proteins, such as ZO-1, Occludins, Claudin-3, and Claudin-5 [32,34–36] are affected. Stimulators, such as LPS, induce the phosphorylation of TJ proteins and myosin light chains, and the recombination of F-actin by activating the mitogen-activated protein kinase (MAPK) and calcium pathways [37]. This contributes to the change in the expression level of TJ proteins on the transcriptional level. Finally, the structure of TJs is destroyed and the permeability of the BBB is disrupted [38,39]. In the present study, the increased BBB permeability and HBMECs monolayers’ permeability were both accompanied by the downregulation of ZO-1, which was significantly reversed by Cabergoline, indicating that Cabergoline repaired the disrupted BBB permeability by restoring the function of TJs.

The classical Wnt/β-catenin pathway in endothelial cells contributes to the development of the BBB during the embryonic period and maintains its integrity and normal function. The inactivity of β-catenin is reported to induce the downregulation of claudin-3 and the degradation of the BBB. In the primary BMECs, the monolayers’ integrity can be increased by the treatment with β-catenin or Wnt3a. However, the disruption of monolayers’ integrity can be induced by inhibiting the activity of the Wnt/β-catenin pathway [40–42]. In the present study, we found that the Wnt/β-catenin pathway in HBMECs was significantly suppressed by LPS but activated by Cabergoline. We suspected that Cabergoline repressed the activity of the natural inhibitor of the Wnt/β-catenin pathway, DKK-3, which is reported to exert significant antitumor efficacy by suppressing the activity of the Wnt/β-catenin pathway in multiple types of malignant tumors [43,44]. We found that Cabergoline significantly reversed the upregulated DKK-3 in LPS-treated HBMECs. We further established the DKK-3-overexpressed HBMECs to verify the mechanism of Cabergoline. We found that the alleviated endothelial monolayer’s permeability, activated Wnt/β-catenin pathway, and increased expression level of ZO-1 in LPS-treated HBMECs induced by Cabergoline were all abolished by the overexpression of DKK-3, indicating that Cabergoline exerted its protective effect on BBB permeability by downregulating DKK-3. Interestingly, brain-protective effects of Cabergoline have been reported in previous studies. Firstly, Cabergoline has been widely used for the treatment of Parkinson’s disease and hyperprolactinemia. Additionally, it was reported to prevent neuronal cell death under oxidative stress via a dopamine D2 receptor-mediated mechanism that blocks the extracellular regulated protein kinases (ERK) signaling pathway, which is well known to play an important part in neuroprotection [45]. However, it should be noted that the underlying mechanism still needs to be elucidated. Firstly, although the dopamine D2 receptor is known to be expressed in endothelial cells [20], it’s still unknown whether the beneficial effects of Cabergoline are dependent or independent of it. Additionally, the interaction between DKK3 and Cabergoline should be further clarified in our future studies.

## Conclusion

Taken together, our data reveals that Cabergoline possessed a protective effect in both *in vivo* mice and *in vitro* cells models induced with LPS, indicating that it could preserve BBB integrity against LPS.

## Data Availability

The data and materials of this study are available upon reasonable request from the corresponding authors.
